# Surgical management of an aneurysmal coronary cameral fistula to the right atrium

**DOI:** 10.21542/gcsp.2021.28

**Published:** 2021-12-31

**Authors:** Andrew M. Acker, Michael E. Ibrahim, Michael A. Acker

**Affiliations:** University of Pennsylvania, Division of Cardiovascular Surgery, Department of Surgery, Philadelphia, Pennsylvania, United States of America

## Abstract

Coronary artery aneurysm and coronary cameral fistula are both often incidentally discovered, and uncommon diagnoses. A coronary artery aneurysm is defined as being 1.5x the size of the adjacent normal coronary artery, while a coronary cameral fistula is a coronary artery that has an abnormal tract directly into a chamber of the heart. In this case report we describe the case of a 72-year-old female who was discovered to have an aneurysmal branch of her right coronary artery fistulized to her right atrium. The fistula had a serpiginous tract and in the midportion of the tract there was a giant aneurysm (3.4 × 3.3 × 3.0 cm) before emptying into the posterior aspect of the right atrium. The aneurysmal aberrant coronary artery was repaired by oversewing the afferent and efferent limbs from the inside. The aneurysm walls were then oversewn. This case demonstrates a unique pathology that was managed with surgical intervention where no standard therapy exists.

## Introduction

Coronary cameral fistula (CCF) is an abnormal communication between a coronary artery and a chamber of the heart. It is an uncommon congenital anomaly seen in 0.9% of computed tomographic angiography (CTA) studies. Pooled data suggest that CCF arises from the RCA in 50–55% of cases, left anterior descending (LAD) in 35–40%, and left circumflex (LCX) in 5–15%^1^. The vast majority communicate with a low pressure right-sided chamber. They are generally asymptomatic and intervention is not recommended unless there is significant shunting of blood, symptoms consistent with myocardial ischemia, or congestive heart failure (CHF)^2^.

Surgical intervention, either by a transcatheter approach or open surgery, is recommended for large diameter CCF, without symptoms, or it is recommended for any sized CCF if symptoms are present^3^. Signs and symptoms are non-specific and include myocardial ischemia, arrhythmia, ventricular systolic or diastolic dysfunction or ventricular enlargement. Diagnostic techniques include CTA or coronary angiography. Surgical management involves either ligation of the culprit vessel, ligation proximally and distally followed by coronary artery bypass grafting (CABG), or internal closure of the fistula efferent site.

Coronary artery aneurysm (CAA) is also an uncommon pathology first discovered in 1812 by French physician, Dr. Charles Bougon^4^. It is defined as a focal dilation of at least 1.5 times the adjacent normal coronary artery. It is now known to have an incidence ranging from 0.3−5.0%, affects proximal coronaries more than distal, and affects the RCA (40%) more frequently than the LAD (32%) and the LCX (23%)^5^.

There are many potential eitiologies of CAA including vasculitic, genetic, athersclerotic, and post-coronary artery bypass saphenous vein grafting. They can be saccular, where the transverse diameter exceeds the longitudinal, or fusiform, where the opposite is true. They can form true aneurysms where all three layers of the vessel are involved or pseudo-aneurysms. The methods for detection include coronary angiography, intravascular ultrasound (IVUS), coronary CT, and functional flow reserve (FFR). The management of this disease process is not standardized and involves medical management, percutaneous intervention, and open surgical correction^6,7,8^.

### Case report

We present the case of a 72-year-old female with a history of osteoporosis, palpitations, and supraventricular tachycardia (SVT) diagnosed 10 years prior. She presented 10 years ago with symptoms of dizziness and light headedness, which required an initial chest X-ray (CXR) and electrocardiogram (ECG), followed by subsequent Holter monitor use, which revealed little. Since that time, she had intermittent self-limiting bouts of SVT.

One year prior she had routine cardiology follow up where she had a surveillance echocardiogram showing a left ventricular ejection fraction (LVEF) of 60%, diastolic and systolic diameters of 4.48 cm and 3.15 cm respectively, mild mitral regurgitation (MR), trace tricuspid regurgitation (TR) and the presence of a patent foramen ovale (PFO). Due to persistent episodes of SVT she was recommended a cardiac catheterization, but the patient requested a less invasive test. She therefore underwent a coronary CTA which reported no coronary artery disease (CAD), but did show the presence of a large aberrant vessel coming off the right coronary artery (RCA), fistulizing to the right atrium (RA).

**Figure 1.**
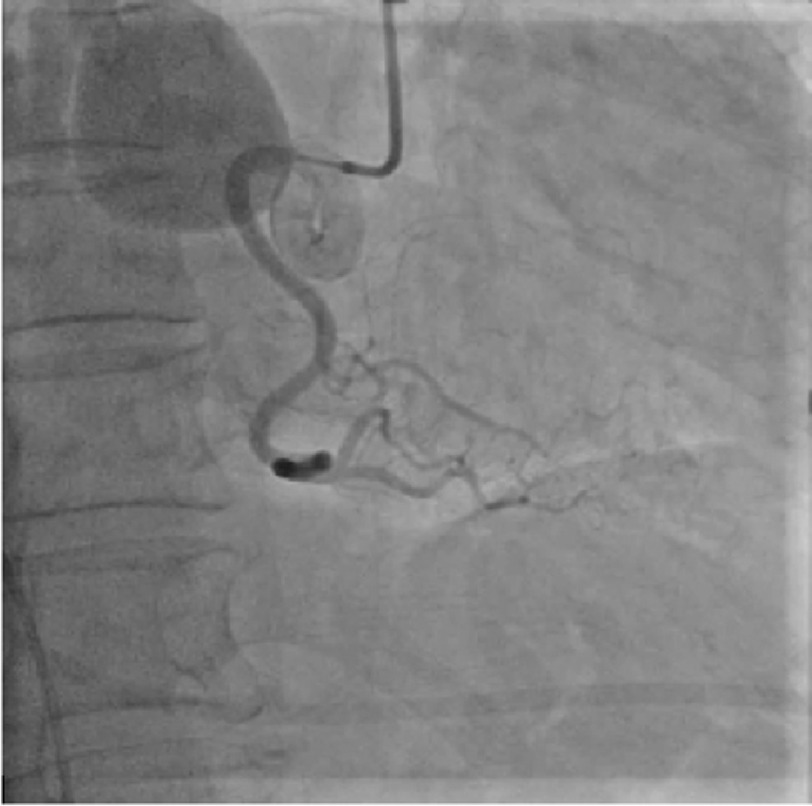
Coronary angiography of the right coronary artery demonstrating an aberrant aneurysmal branch draining into the right atrium.

It originated in the proximal atrioventricular groove extending superiorly and posteriorly. The fistula had a serpiginous tract, which has a giant aneurysm measuring 3.4 × 3.3 × 3.0 cm in the midportion of the tract. Her workup also consisted of a right heart catheterization (RHC), which demonstrated a RA pressure of 2 mmHg, right ventricular (RV) pressure of 32/2 (19) mmHg, pulmonary capillary wedge pressure (PCWP) of 7 mmHg, pulmonary artery (PA) saturation of 77%, a cardiac output (CO)/cardiac index (CI) of 6/3.8, a pulmonary vascular resistance (PVR) of 1.9, and a small left to right shunt of 1.1 to 1. Coronary angiography was also eventually performed which did not show any CAD, but did corroborate the findings on the coronary CTA (Figure 1).

**Figure 2.**
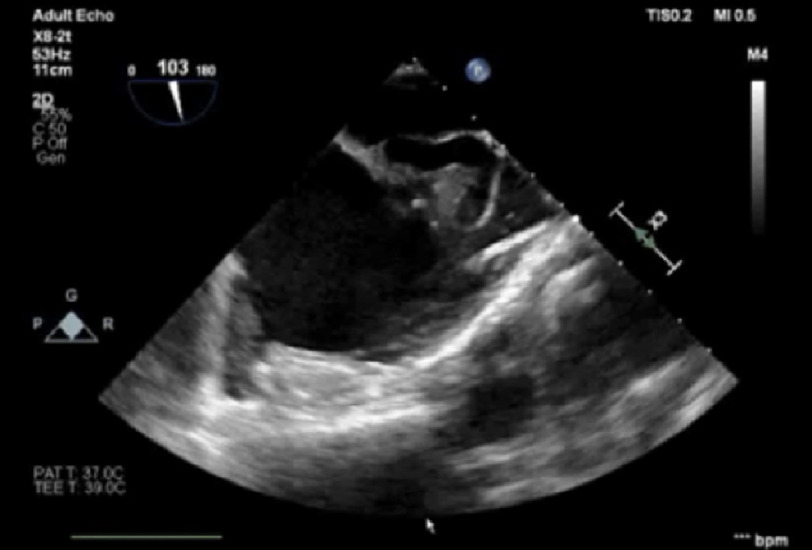
Transesophageal echocardiogram image demonstrating a coronary artery aneurysm of a branch from the right coronary artery.

She was referred for surgical repair. The intra-operative TEE demonstrated the findings described above (Figure 2).

**Figure 3.**
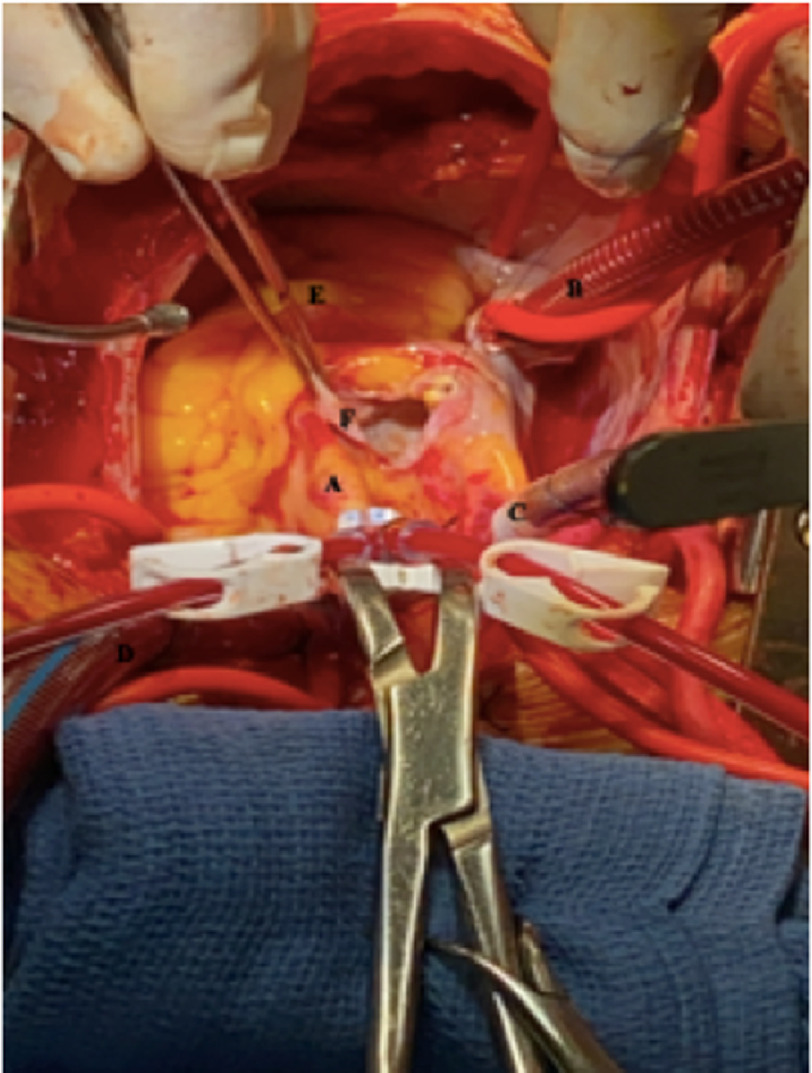
A – Ascending aorta, B – Inferior vena cava cannula, C – Superior vena cava cannula, D – Aortic cannula, E – Right ventricle, F – Opened coronary artery aneurysm.

In the operating room, once standard cardiopulmonary bypass had been established and the heart was arrested, the aneurysm was easily identified in the proximal atrioventricular groove. The aneurysm sac was opened widely (Figure 3).

Cold cardioplegia solution was given through the aortic root in order to visualize the arterial limb of the aneurysm, which was then oversewn using a 4-0 prolene suture. Similarly, by temporarily occluding the venous drainage cannula and filling the RA, the efferent limb of the aberrant aneurysm was visualized and then subsequently oversewn with a 4-0 prolene suture. The aneurysm walls were then reapproximated with a 4-0 prolene suture. No coronary bypass grafting was required and the patient weaned off cardiopulmonary bypass with ease.

She was then transferred to the cardiac surgery intensive care unit (ICU), extubated on post-operative day (POD) 0, and transferred to the step-down unit on POD 1. She was bradycardic with a junctional rhythm post-operatively necessitating dual-chamber permanent pacemaker (PPM). We suspect a small branch feeding the sinoatrial node may have been damaged during repair. She was eventually discharged home in stable condition on POD 9.

One month post-operatively she continued to have episodes of SVT, which required an ablation. At the time of writing this report it has been ten months since her operation and she is functioning well.

### Comment

This case demonstrates a number of features of both CCF and CAA, including their typical course, features on multi-modality imaging, and the difficulty in attributing symptoms to their presence. This case highlights questions that still need to be answered in the field like whether incidentally discovered aneurysms require intervention and what the post-operative medical management ought to be. Given their rarity, it has not been possible to quantify their risk of rupture or dissection. There are no current guidelines on how to manage CAA and CCF; these cases should be approached from an interdisciplinary heart team that includes both cardiac surgery and interventional cardiology to determine the optimal strategy.
